# The Challenge of Return to Work after Breast Cancer: The Role of Family Situation, CANTO Cohort

**DOI:** 10.3390/curroncol28050330

**Published:** 2021-10-01

**Authors:** Elsa Caumette, Inès Vaz-Luis, Sandrine Pinto, Julie Havas, Thomas Bovagnet, Garazi Ruiz de Azua, Antonio Di Meglio, Anne-Laure Martin, Sibille Everhard, Paul Cottu, Laurence Vanlemmens, Christelle Jouannaud, Florence Lerebours, Agnès Dumas, Gwenn Menvielle

**Affiliations:** 1 IPLESP, Équipe de Recherche en Épidémiologie Sociale, INSERM, Institut Pierre Louis d’Épidémiologie et de Santé Publique, Sorbonne Université, 75012 Paris, France; elsa.caumette@umontpellier.fr (E.C.); sandrine.pinto@iplesp.upmc.fr (S.P.); thomas.bovagnet@iplesp.upmc.fr (T.B.); garazi.ruizdeazua@iplesp.upmc.fr (G.R.d.A.); gwenn.menvielle@inserm.fr (G.M.); 2 Department of maieutics, University of Montpellier, 34000 Montpellier, France; 3 Breast Cancer Unit, Department of Medical Oncology, Gustave Roussy, 94800 Villejuif, France; INES-MARIA.VAZ-DUARTE-LUIS@gustaveroussy.fr; 4 INSERM Unit U 981, Gustave Roussy, 94800 Villejuif, France; Julie.HAVAS@gustaveroussy.fr (J.H.); Antonio.DI-MEGLIO@gustaveroussy.fr (A.D.M.); 5 Clinical Research Department, Gustave Roussy, 94800 Villejuif, France; 6 UNICANCER, 75013 Paris, France; al-martin@unicancer.fr (A.-L.M.); s-everhard@unicancer.fr (S.E.); 7 Institute Curie, 75005 Paris, France; paul.cottu@curie.net; 8 Center Oscar Lambret, 59000 Lille, France; l-vanlemmens@o-lambret.fr; 9 Institute Jean Godinot, 51100 Reims, France; christelle.jouannaud@reims.unicancer.fr; 10 Institute Curie, 92210 Saint Cloud, France; florence.lerebours@curie.fr; 11 ECEVE UMR 1123, INSERM (National Institute for Health and Medical Research), Université de Paris, 75010 Paris, France

**Keywords:** return to work, breast cancer, family situation, cohort

## Abstract

Return to work (RTW) after breast cancer is associated with improved quality of life. The link between household characteristics and RTW remains largely unknown. The aim of this study was to examine the effect of the family situation on women’s RTW two years after breast cancer. We used data of a French prospective cohort of women diagnosed with stage I-III, primary breast cancer (CANTO, NCT01993498). Among women employed at diagnosis and under 57 years old, we assessed the association between household characteristics (living with a partner, marital status, number and age of economically dependent children, support by the partner) and RTW. Logistic regression models were adjusted for age, household income, stage, comorbidities, treatments and their side effects. Analyzes stratified by age and household income were performed to assess the association between household characteristics and RTW in specific subgroups. Among the 3004 patients included, women living with a partner returned less to work (OR = 0.63 [0.47–0.86]) and decreased their working time after RTW. Among the 2305 women living with a partner, being married was associated with decreased RTW among women aged over 50 (OR = 0.57 [0.34–0.95]). Having three or more children (vs. none) was associated with lower RTW among women with low household income (OR = 0.28 [0.10–0.80]). Household characteristics should be considered in addition to clinical information to identify vulnerable women, reduce the social consequence of cancer and improve their quality of life.

## 1. Introduction

Breast cancer is the leading cancer in women [1] but it also has one of the best 5-year survival rates (nearly 90%) [2,3]. The high survival rate may be related to the combination of early diagnosis, novel surgical techniques, and new medications [4,5]. In France, in 2018, 58,400 women were diagnosed with breast cancer, about half of them were between 20 and 59 years of age [6]. A large proportion of women are therefore working when diagnosed, and cancer can have an impact on their work life [7,8]. Furthermore, studies showed that the loss of work after cancer could affect women’s quality of life as well as financial situation [9,10,11] and that return to work (RTW) was seen by women as an important part of their recovery. Understanding all determinants of RTW is thus essential to help patients build a new normality through work and reduce the burden of breast cancer in survivors’ life [12]. Many studies showed that clinical characteristics (stage, comorbidities, treatments and their side effects) were major determinants of RTW after cancer. An advanced stage of cancer, poor health at diagnosis, but also some treatments (e.g., chemotherapy and lymph node dissection) and their physical (e.g., lymphedema of the arm, arm pain and fatigue) and psychological (e.g., depression and anxiety) sequelae have a negative impact on women’s RTW after breast cancer [13,14,15,16]. Favourable working conditions (e.g., non-manual work and support from colleagues) have a positive impact on RTW [17].

Although included in the REWORK-BC model, a theoretical model explaining the RTW process after breast cancer [18], few studies investigated association between household characteristics and RTW. First, living with a partner could be negatively associated with RTW if the partner can ensure the household’s financial security alone [19]. This security could be reinforced by marriage [20]. However, living with a partner could also be positively associated with RTW if the partner provides emotional support, which helps the woman returning to work [21,22]. The studies investigating the association between patients’ relationship status and RTW reported heterogeneous results [16,23]. Furthermore, the definition of couple differed between studies. Some differentiated between women living with a partner (without taking into account women’s marital status) and women not living with a partner [16]. Others including a meta-analysis differentiated between married women and unmarried women (without considering whether the women lived with a partner) [23]. These classifications mixed the concepts of couplehood and marriage and therefore did not enable one to distinguish the impact on RTW of living with a partner from that of being married.

In addition to couplehood, the presence of children, which constitutes a financial burden but also possible support, could impact RTW with possible differences according to the number and age of the children. Several recent studies, including a meta-analysis, showed that having economically dependent children had a positive impact on RTW [23,24]. However, the number of dependent children or their age was seldom taken into account. In particular, no study specifically looked at young children, who could nevertheless be a particular burden because of their low level of autonomy. The literature nevertheless did not suggest any differences, in terms of RTW, between women with or without minor dependent children [25]. 

Finally, several studies explored family support as a facilitator of RTW, but few specifically evaluated partner’s support [22,26]. 

Age and socioeconomic position (SEP) are associated with RTW, with lower RTW among older [27] and low SEP (low level of education, manual work or low income) [28,29] women. These characteristics could influence the impact of household factors on RTW. Indeed, over the past decades, we observed in western countries delayed age at marriage and increased rate of second couplehood [30,31]. The association between couplehood, marriage and RTW may therefore differ by age. In addition, young children could be a barrier to RTW in particular among low SEP women because of the costs related to child care. Similarly, having a partner could be more closely associated with RTW among low SEP women, as complete wage could be more essential when the household income is low. 

Overall, the link between household characteristics and RTW after breast cancer remains largely unknown and most studies on this topic used imprecise variables to characterize household because this was not their main aim. The objective of this study was to examine the effect of family situation on women’s RTW two years after breast cancer and to assess whether these associations differed by patient’s age or household income.

## 2. Materials and Methods

We used data from a French prospective cohort of women diagnosed with stage I-III first primary breast cancer (CANTO). Data collection started in 2012 at 26 cancer care centers. The study was approved by the French regulatory authorities (14 September 2011) and the French Ethics Committee (14 October 2011). All the patients enrolled in the study were aged 18 or over and provided written informed consent. Women were enrolled by an oncologist at breast cancer diagnosis. Data were collected prospectively during a clinical examination and through self-reported questionnaires at diagnosis, about 1 year after diagnosis (the visit took place 3 to 6 months after the end of treatment), and 2 years after diagnosis.

**Figure 1:**
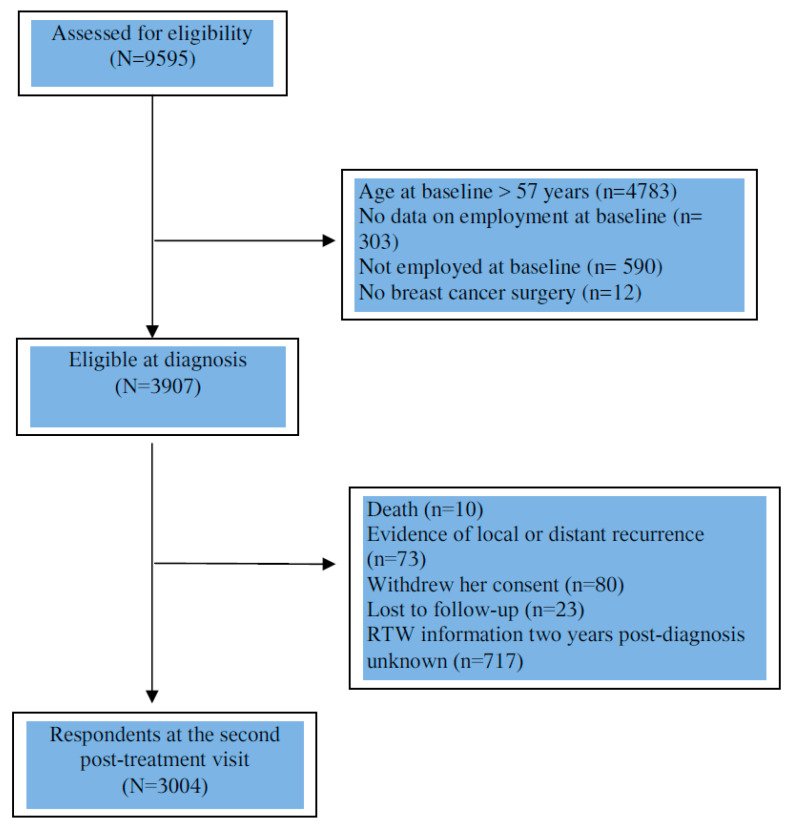
Flow chart of patient population.

The analysis was based on women included until February, 2017. We limited the analysis to women under 57 years of age who were working at the time of diagnosis (*n* = 3919). We excluded women who were not treated with curative intent (patients with no surgery) (*n* = 12), as well as women with evidence of local or distant recurrence (*n* = 73), who withdrew consent (*n* = 80), lost to follow-up (*n* = 23) or deceased (*n* = 10) two years after diagnosis. Then, we excluded women whose RTW information two years post-diagnosis was unknown (*n* = 717). Finally, we included 3004 patients (Figure 1).

The outcome was RTW two years after BC diagnosis and was collected by an ad hoc self-reported questionnaire. We investigated the association between RTW and various household characteristics self-reported at diagnosis in two populations: among all women and restricted to women living with a partner. In addition, we performed analyses stratified by the women’s age (under vs. over 50 years) and household income (below vs. above 2500 euros). Among all women, we studied the fact of living with a partner (yes/no) and the household structure combining the presence of a partner and economically dependent children (single/partnered women without/with children). Economically dependent children were defined as children aged below 25 living in the household. Among women living with a partner, we studied marital status (married/not married), perceived support by the partner(very strong/a little, not enough, not at all) as well as the number of economically dependent children (0, 1, 2, 3 or more) and their age. The impact of the age of children on RTW was assessed in a supplementary analysis. We identified women having children younger than 7 years of age (yes/no), as this age group is more dependent on adults [32], and women having children aged 18–25 (yes/no), as these could be graduate students with important financial needs.

Multivariate logistic regression analyses were performed. The models were adjusted for age (<40, 40–49 or ≥50 years), clinical factors, treatment side effects at the first post-treatment visit (1 year after diagnosis) and household income (<2500, 2500–3000, 3000–4000, >4000 euros).

Clinical factors included stage at diagnosis (I, II, III), comorbidities at diagnosis, treatment and their side effects 1 year after diagnosis. Comorbidities at diagnosis were evaluated using the Charlson comorbidity index [33] (0, 1 ≥ 2) and a binary variable assessing the presence of ≥3 additional comorbid medical conditions not captured by the Charlson. Information about breast surgery (conservative/mastectomy), lymph node surgery (no or sentinel dissection/axillary dissection), systemic treatment (chemotherapy, hormone therapy, anti HER2 therapy) (yes/no) and radiotherapy (yes/no) was collected. Physical side effects of treatment were collected by a nurse, and defined as the presence of any severe toxicities(grade ≥ 3) using version 4 of the Common Toxicity Criteria Adverse Events Scale (CTCAE) [34]. Additional treatment side effects were collected using three subscales of the breast cancer module (QLQ-BR23) (systemic therapy side effects, arm morbidity and breast morbidity as continuous variables) of the European Organization for Research and Treatment of Cancer (EORTC) self-reported quality-of-life questionnaire and the fatigue subscale (as continuous variable) of the core EORTC questionnaire(QLQ-C30) [35]. In addition, anxiety and depression were assessed using the Hospital Anxiety and Depression Scale (HADS) (non-case (0–7), doubtful (8–10), case (11–21) [36]. 

A sensitivity analysis was realized in the subgroup of women who returned to work and with known working conditions, to investigate the association between household characteristics and change in working time between diagnosis and two years after diagnosis using multinomial logistic regressions (full-time work two years after diagnosis, full-time work at diagnosis and part-time work two years after diagnosis, always part-time work). All analyses were performed with SAS 9.4 (SAS software for Windows version 9.4, SAS Institute Inc., Cary, NC, USA).

## 3. Results

In this study, the median age of women was 48 years (between 23 and 56 years). Women had 78% of the women lived with a partner and 11% were single mothers (Table 1). Most of the women living with a partner were married (73%) and received very strong support from their partner (85%). Furthermore, among women living with a partner, most women had dependent children (72%), 14% had children younger than 7 years of age and 32% had children aged 18–25. 

RTW two years after breast cancer diagnosis in relation to clinical characteristics was described in Table S1. About two thirds of the women worked full-time two years after diagnosis (regardless of their work time (full-time or part-time)at diagnosis), 17 % changed from a full-time to a part time job and 18% were still working part-time.

Among all women (Table 2), living with a partner was associated with lower RTW (OR = 0.63 [0.47–0.86]) and with a decrease in working time after RTW (OR = 1.6 [1.00–2.45] compared to full-time work two years after diagnosis (Table S2). RTW was increased for single women without dependent children (OR = 1.62 [1.07–2.47]) compared to women living with a partner and having dependent children.

Among women living with a partner (Table 3), being married was associated with decreased RTW only in those over age 50 (OR = 0.57 [0.34–0.95]). Having any (versus no) dependent children was associated with lower RTW only in the women with three or more children and in the low household income women (OR = 0.28 [0.10–0.80]). RTW also differed according to the age of economically dependent children. The results suggested lower RTW among women having any (*N* = 409) (versus no) dependent children under 7 years of age (OR = 0.73 [0.49–1.08]) and higher RTW among women having any (*N* = 736) (versus no) dependent children aged 18–25 (OR = 1.21 [0.91–1.60]). Perceived support by the partner was not associated with RTW either for the entire sample or in the age- or household income-stratified analyses.

## 4. Discussion

The aim of this study was to examine the effect of family situation (living with a partner and his support, being married, having children) on women’s RTW and work time two years after breast cancer diagnosis. 

There is no general consensus in the literature on the association between the presence of a partner and RTW after breast cancer [16,23,37]. In this study, at equal household income at diagnosis, living with a partner appeared to decrease RTW but also working time after RTW. This points to the importance of the financial dimension and the partner financial support for RTW through more freedom and flexibility in the process of RTW.

In addition to couplehood, we were able to study the specific effect of marriage on RTW after breast cancer in women living with a partner, which is almost never done in the literature [22]. In this study, among women living with a partner, marriage was generally not associated with RTW, but married women returned less to work than non-married women in the women over 50 years of age. Among women living with a partner and over 50 years of age most were married (79%) and married women had a statistically longer couple duration than the unmarried ones (median 30 years vs. 15 years, difference statistically significant). This is consistent with what is observed in the general population. Unmarried women over 50 years of age are therefore more likely to be in a recent relationship and consequently may have more psychological and financial independence, which would explain a higher rate of RTW among these women [38].

In the literature, there is a positive association between having dependent children and RTW after breast cancer [23]. The results of this study only partly support these findings. First, this analysis on the household structure and RTW among all women emphasizes the lack of association between the presence of economically dependent children and RTW. As regards partnered women, we found that having dependent children had a negative effect on RTW, but only among the women with at least three children and a low household income. In the general population, a threshold effect is observed in the association between the number of children and the employment rate: women work much less from three children upwards [39]. The negative effect of having more than three children and a low SEP on RTW could thus sum up and constitute an important barrier to RTW.

In the scarce literature taking into account the children’s age, a study did not find any association between having minor dependent children and RTW [25]. A German cohort study nevertheless observed that having children decreased RTW among women between 18 and 44 years of age compared to older women [24], suggesting that young children may be a barrier to returning to work. This is consistent with our findings. Although based on small numbers, we observed a tendency towards lower RTW among women with children below age 7 and increased RTW among women having children aged 18–25. The latter finding may be related to the need for money associated with the fees of graduate studies.

Theoretical models such as the REWORK-BC model already suggested the importance of household characteristics in RTW process after breast cancer [18] but this study provides new insights into the role of household characteristics on RTW thanks to the large sample size, the detailed information collected on household variables, and the inclusion of a large number of confounding factors (SEP, age, clinical factors, treatment and their side-effects). Then, while most studies only investigate RTW, we also studied change in working time after RTW. Nevertheless, several limitations should be discussed. First, perceived support by the partner was collected only at the time of diagnosis whereas it can vary during the course of the disease and should therefore be better assessed over time. This may explain why we did not find partner’s moral support to be associated with RTW. Indeed, in the literature, the qualitative [40] and quantitative [22] studies that mentioned partner’s support as a facilitator of RTW after breast cancer measured this parameter over the long term (e.g., 3 to 4 years after diagnosis). Secondly, 19% of the eligible participants did not answer to the question on RTW, which could have biased the results of the study. Missing information on RTW was significantly less frequent for women living with a partner, after 40 years old, with a higher household income, and treated by hormonotherapy. Therefore, the proportion of women living with a partner could be slightly overestimated.

For most patients, cancer is a physical and emotional earthquake in their life [41]. The RTW period is often seen as the first step towards a new normal life [42]. The results of this study identified populations who were less likely to RTW. These patients could be at risk for poorer quality of life and psychosocial wellbeing. It is therefore essential that clinicians, as the first contact with patients, identify and refer them to social workers early in the disease process, therefore reducing the burden of breast cancer on women’s life.

## 5. Conclusions

RTW is a complex social process where clinical, demographic and social variables interact. In addition to women’s clinical characteristics, demographic household characteristics are important determinants for RTW after breast cancer diagnosis and working time after RTW. These associations differ by age and household income. Further studies investigating the reasons to stop working would help better understand the barriers for RTW.

## Figures and Tables

**Table 1:** Household demographic characteristics and proportion of women who returned to work two years after early breast cancer diagnosis.

	*N* (%) ^†^	% RTW
Entire study population (*N* = 3004)		
Living with a partner		
Yes	2305 (78.5)	79.5
No	632 (21.5)	79.9
Missing	67	
Household structure		
Single woman with no economically dependent children	284 (9.7)	80.3
Partnered woman with no economically dependent children	646 (22.2)	76.5
Single mother	333 (11.4)	79.9
Partnered woman with economically dependent children	1653 (56.7)	80.7
Missing	88	
Women living with a partner (*N* = 2305)		
Marital status		
Married	1675 (73.0)	79.0
Not married	618 (27.0)	81.1
Missing	12	
Number of economically dependent children		
0	646 (28.1)	76.5
1	524 (22.8)	79.0
2	833 (36.2)	82.4
3 or more	296 (12.9)	79.1
Missing	6	
Support by the partner		
Very strong	1930 (84.7)	79.4
A little, not enough, not at all	348 (15.3)	80.5
Missing	27	

*N*: Number of patients; RTW: Return to work. ^†^ The percentages do not include the missing data.

**Table 2:** Odds ratios (ORs) and their 95% confidence intervals (CIs) between household demographic characteristics and return to work two years after early breast cancer diagnosis in all women by their age and household income (*N* = 2347).

	All Women	Analyses by Age Group		Analyses by Monthly Household Income	
	*N* = 2347	<50 Years of Age *N* = 1412	≥50 Years of Age *N* = 935	<2500 Euros *N* = 673	≥2500 Euros *N* = 1674
	OR ^†^ [95% CI]	OR ^†^ [95% CI]	OR ^†^ [95% CI]	OR ^†^ [95% CI]	OR ^†^ [95% CI]
Living with a partner					
No	1	1	1	1	1
Yes	0.63 [0.47–0.86]	0.71 [0.47–1.08]	0.55 [0.35–0.88]	0.68 [0.47–1.00]	0.56 [0.32–0.99]
Household structure					
Single woman with no economically dependent children	1.62 [1.07–2.47]	1.49 [0.82–2.73]	1.77 [0.96–3.25]	1.7 [0.98–2.84]	2.23 [0.88–5.65]
Partnered woman with no economically dependent children	0.90 [0.68–1.20]	1.04 [0.65–1.66]	0.84 [0.57–1.24]	1.22 [0.67–2.21]	0.81 [0.58–1.12]
Single mother with economically dependent children	1.45 [0.98–2.13]	1.38 [0.85–2.25]	1.47 [0.76–2.86]	1.48 [0.90–2.45]	1.37 [0.68–2,76]
Partnered woman with economically dependent children	1	1	1	1	1

^†^ Models additionally adjusted for age (except in the analyses stratified by age), household income (except in the analyses stratified by household income), stage at diagnosis, health at diagnosis (Charlson score, other medical antecedents), treatment(chemotherapy, hormone therapy, anti HER2 therapy, radiotherapy, breast surgery, lymph node surgery), health one year after diagnosis (fatigue, anxiety, depression, arm morbidity, breast morbidity, systemic therapy side effects, and severe physical toxicities).

**Table 3:** Odds ratios (ORs) and their 95% confidence intervals (CIs) between household demographic characteristics and return to work two years after early breast cancer diagnosis in partnered women according to age and household income.

	All Partnered Women	Analyses by Age Group		Analyses by Monthly Household Income	
	*N* = 1817	<50 Years of Age *N* = 1100	≥50 Years of Age *N* = 717	<2500 Euros *N* = 314	≥2500 Euros *N* = 1503
	OR ^†^ [95% CI]	OR ^†^ [95% CI]	OR ^†^ [95% CI]	OR ^†^ [95% CI]	OR ^†^ [95% CI]
Marital status					
Not married	1	1	1	1	1
Married	0.79 [0.59- 1.06]	0.92 [0.64–1.33]	0.57 [0.34- 0.95]	0.63 [0.34–1.18]	0.88 [0.63–1.23]
Number of economically dependent children					
0	1	1	1	1	1
1	1.02 [0.72–1.44]	0.76 [0.44–1.34]	1.30 [0.80–2.09]	1.03 [0.46–2.28]	1.02 [0.69–1.51]
2	1.25 [0.88–1.77]	1.11 [0.66–1.9]	1.17 [0.69–2.00]	0.65 [0.28–1,48]	1.42 [0.96–2.10]
3 or more	0.9 [0.64–1.55]	0.79 [0.44–1.42]	1.34 [0.48–3.76]	0.28 [0.10–0,80]	1.33 [0.79–2.23]
Perceived support by the partner					
A little, not enough, not at all	1	1	1	1	1
Verystrong	0.83 [0.58–1.19]	0.72 [0.43–1.21]	1.05 [0.62–1.76]	0.52 [0.24–1.11]	0.97 [0.64–1.48]

^†^ Models additionally adjusted for age (except in the analyses stratified by age), household income (except in the analyses stratified by household income), stage at diagnosis, health at diagnosis (Charlson score, other medical antecedents), treatment(chemotherapy, hormone therapy, anti HER2 therapy, radiotherapy, breast surgery, lymph node surgery), health one year after diagnosis (fatigue, anxiety, depression, arm morbidity, breast morbidity, systemic therapy side effects, and severe physical toxicities) and the other two sociodemographic variables.

## Data Availability

Deidentified participant data available under request to control the results of the study (contact the corresponding author, agnes.dumas@inserm.fr)The CANTO data are open to the scientific community. Any project can be submitted. Please contact the promotor Unicancer for further information: s-everhard@unicancer.fr.

## Supplementary Materials

The following are available online at https://www.mdpi.com/article/10.3390/curroncol28050330/s1, Table S1: Clinical characteristics and proportion of women who returned to work two years after early breast cancer diagnosis, Table S2: Odds ratios (ORs) and their 95% confidence intervals (CIs) between household demographic characteristics and evolution in working time between diagnosis and two years after early breast cancer diagnosis in all women and in partnered women.
