# Supplementary material for: The Challenge of Return to Work after Breast Cancer: The Role of Family Situation, CANTO Cohort
Source: Curr Oncol. 2021 Oct 1;28(5):3866–75. doi: 10.3390/curroncol28050330 (PMC8534983; doi:10.3390/curroncol28050330)
Supplement: Supplementary file 1 [file curroncol-28-00330-s001.zip › curroncol-1400700-supplementary.pdf]

Table S1. Clinical characteristics and proportion of women who returned to work two years after early breast cancer diagnosis

	All women †	Women who returned to work
**Age(years), N (%)**		
18-39	389 (13.0)	81.0
40-49	1389 (46.2)	81.4
50-56	1226 (40.8)	77
Missing	0	
**Charlson index at diagnosis, N (%)**		
0	2430 (86.7)	80.9
1	212 (7.6)	69.3
≥2	160 (5.7)	73.1
Missing	202	
**Additional comorbid medical conditions at diagnosis, not captured by the Charlson, N (%)**		
<3	2362 (79,5)	81.2
≥3	608 (20.5)	72.9
Missing	34	
**Stage at diagnosis, N (%)**		
Stage I	1330 (44.3)	84.1
Stage II	1341 (44.6)	77.4
Stage III	333 (11.1)	70.3
Missing	0	
**Breast surgery, N (%)**		
Conservative	2107 (70.1)	82.9
Mastectomy	897 (29.9)	71.8
Missing	0	
**Lymph node surgery, N (%)**		
None or sentinel node dissection	1718 (57.2)	83.4

Axillary dissection	1286 (42.8)	74.4
Missing	0	
**Chemotherapy, N (%)**		
No	1084 (36.1)	84.8
Yes	1920 (63.9)	76.6
Missing	0	
**Hormotherapy, N (%)**		
No	532 (17.7)	75.8
Yes	2471 (82.3)	80.4
Missing	1	
**Trastuzumab, N (%)**		
No	2561 (85.3)	80.8
Yes	442 (14.7)	72.2
Missing	1	
**Radiotherapy, N (%)**		
No	218 (7.3)	73.9
Yes	2785 (92.7)	80
Missing	1	
**Severe physical toxicities one year after diagnosis, N (%)**		
0	2701 (90.9)	81.0
≥1	271 (9.1)	66.4
Missing	32	
**Systemic therapy side effects subscale‡ one year after diagnosis, median (Q1-Q3)**	14.3 (9.5-28.6)	14,3 (9.5-23.8)
**Arm morbidity subscale‡ one year after diagnosis, median (Q1-Q3)**	16.7 (0-50)	16,7 (0-33.3)
**Breast morbidity subscale‡ one year after diagnosis, median (Q1-Q3)**	25 (16.7-41.7)	25 (16.7-33.3)
**Fatigue subscale‡ one year after diagnosis, median (Q1-Q3)**	33.3 (22.2-55.6)	33,3 (22.2-55.6)
**Anxiety§ one year after diagnosis, N (%)**		
Non-case	1469 (53.0)	85.0
Doubtful	710 (25.6)	75.8

Case	595 (21.5)	70.8
Missing	230	
**Depression[c] one year after diagnosis, N (%)**		
Non-case	2305 (83.2)	82.7
Doubtful	298 (10.8)	69.8
Case	168 (6.6)	54.8
Missing	233	

N Number of patients; *RTW* Return to work.

† The missing data do not included for the percentages

‡ Additional treatment side effects were collected using three subscales of the breast cancer module (QLQ-BR23) (namely, systemic therapy side effects, arm morbidity and breast morbidity) of the European Organization for Research and Treatment of Cancer (EORTC) self-reported quality-of-life questionnaire and the fatigue subscale of the core EORTC questionnaire(QLQ-C30)

§ Anxiety and depression were assessed using the Hospital Anxiety and Depression Scale (HADS). Both subscales were divided into three categories (non-case [0-7], doubtful [8-10] and case [11-21])

Table S2. Odds ratios (ORs) and their 95% confidence intervals (CIs) between household demographic characteristics and evolution in working time between diagnosis and two years after early breast cancer diagnosis in all women and in partnered women

	Full time 2 years after diagnosis	Decrease from full time to part time	Always part time
	OR	OR [95% CI]	OR [95% CI]
**All women (N=1248)**			
Living with a partner (reference No)			
Yes	1	**1,56 [1.00-2,45]**	**4,31 [2,55-7,29]**
Household structure (reference Single woman with no economically dependent children)			
Partnered woman with no economically dependent children	1	**2,01 [1,01-3,98]**	**6,90 [2,55-18,68]**
Single mother with economically dependent children	1	1,68 [0,84-3,37]	**3,41 [1,20-9,74]**
Partnered woman with economically dependent children	1	**2,15 [1,14-4,06]**	**10,83 [4,15-28,24]**

**Partnered women (N=970)**			
Marital status(reference Not married)			
Married	1	1,05 [0,70-1,57]	**1,47 [0,98-2,20]**
Number of economically dependent children (reference 0)			
1	1	1,00 [0,59-1,69]	1,04 [0,61-1,75]
2	1	0,95 [0,57-1,57]	**1,63 [1,01-2,65]**
3 or more	1	0,82 [0,42-1,63]	**2,54 [1,41-4,57]**
Perceived support by the partner (reference A little, not enough, not at all)			
Very strong	1	1,03 [0,62-1,72]	0,98 [0,62-1,54]

All Model were adjusted for age, household income, stage at diagnosis, health at diagnosis (Charlson score, other medical antecedents), treatment (chemotherapy, hormone therapy, anti HER2 therapy, radiotherapy, breast surgery, lymph node surgery), health one year after diagnosis (fatigue, anxiety, depression, arm morbidity, breast morbidity, systemic therapy side effects, and severe physical toxicities).
Analyses among partnered women were additionally adjusted for marital status, the number of economically dependent children and perceived support by partner.
